# Context effects: discourse structure influences narrative ability in autism and first-degree relatives

**DOI:** 10.3389/fpsyt.2025.1588429

**Published:** 2025-09-04

**Authors:** Emily Landau, Kritika Nayar, Gary E. Martin, Cassandra Stevens, Jiayin Xing, Janna Guilfoyle, Joseph C. Y. Lau, Molly Losh

**Affiliations:** ^1^ Roxelyn and Richard Pepper Department of Communication Sciences and Disorders, Northwestern University, Evanston, IL, United States; ^2^ Department of Child and Adolescent Psychiatry, Hassenfeld Children’s Hospital at NYU Langone, New York City, NY, United States; ^3^ Department of Communication Sciences and Disorders, St. John’s University, Queens, NY, United States; ^4^ Schiefelbusch Institute for Life Span Studies and Kansas Center for Autism Research and Training, University of Kansas, Lawrence, KS, United States

**Keywords:** autism, siblings, broad autism phenotype, narrative, eye tracking, storytelling

## Abstract

**Introduction:**

Narrative, or storytelling, ability is a well-documented area of difficulty in autism spectrum disorder (ASD) and is an important skill that is related to social-communicative success. Evidence also demonstrates subtle narrative differences among first-degree relatives of autistic individuals, including parents (ASD parents) and siblings (ASD siblings), suggesting narrative ability may reflect genetic influences related to ASD. Less structured contexts, such as free form narrative retellings (i.e., without scaffolding via visual aids), require individuals to reconstruct a previously told narrative from memory and reflect differences in underlying attention, language, and executive functioning. Narrative retellings are impacted in ASD, though work has yet to examine this ability in first-degree relatives. A prior study employed a first telling narrative task (First Telling) involving simultaneous viewing of a picture book among autistic individuals, their parents, siblings, and respective control groups while collecting eye tracking data to extrapolate attentional mechanisms.

**Methods:**

The present study aimed to extend this work by adding an additional less structured narrative retelling (Retell task) to characterize the breakdown in narrative quality between different contexts and assess how narration and visual attention during the First Telling narrative may relate to narrative quality in the Retell task.

**Results:**

As predicted, narrative retellings were less sophisticated than first-telling narratives, and the quality of the First Telling was related to the quality of the Retell narrative for all groups. Some overlapping patterns of narrative quality emerged between individuals with ASD, their parents, and siblings. No associations emerged between visual attention in the First Telling and narrative quality in the Retell task.

**Discussion:**

Results support previous findings of narrative challenges in ASD and provide evidence that narrative skills may be subtly impacted in first degree relatives, suggesting ASD-related genetic influence on elements of narrative ability. Findings may inform intervention efforts, as the lack of visual supports in the retell task impacted narrative quality in ways that parallel the challenges individuals may face in everyday storytelling and naturalistic conversational interactions.

## 1 Introduction

Challenges with social communication are a hallmark feature of autism spectrum disorder (ASD) (1), including differences in narrative ability (i.e., storytelling) (e.g., 2).^1^ Narrative refers to communication of events rendered from a particular perspective that are linked temporally and causally, and are strongly bound to cultural conventions (3, 4). Narrative is often used to interpret the intentions of others and determine meaning. Consequently, narrative ability is essential in the development of meaningful relationships, as it contributes to important pragmatic (i.e., social) skills that are the bedrock of building connections with others (5, 6). Narrative ability also plays a key role in memory formation and consolidation, shaping an autobiographical sense of self, and organizing experiences into meaningful stories, while also supporting critical developmental skills such as academic success, language development, and emotional understanding (7–22). Thus, fluid narrative ability—encompassing the description of experiences, relating them to others, and forming a sense of self—is crucial for social-communicative success (13).

Documented challenges in narrative ability in autistic individuals include reduced narrative coherence (e.g., connections between story elements) (23–26), fewer inferred causal relationships (e.g., stating why a character acted or felt a certain way) (23, 27, 28), less frequent references to characters’ emotional and cognitive internal states (e.g., stating that a character feels happy) (27–31), and increased use of off-topic remarks (27, 32–35), both in highly structured storybook contexts, as well as more naturalistic conversational interactions where narratives commonly occur. Additionally, narrative ability in autistic individuals has been found to relate to social cognition, suggesting that difficulty understanding another’s point of view may impact the ability to adequately narrate and infer another’s emotional and cognitive states (23, 27, 36, 37).

Many studies of narrative used wordless storybooks or complex static images to elicit narratives, where participants view the images and describe the story (e.g., 23, 25–28, 30, 31, 37–39). Other studies have used a less-structured narrative retelling context, where participants retell the story to an examiner after a delay in viewing images (e.g., 24, 26, 33, 34, 39–46). Narrative retellings can be employed to increase cognitive demand by requiring reconstruction of a previously encoded story, providing insight into how an individual attended to, processed and organized, and remembered their initial narrative (47–49). Retellings may be more similar to narratives occurring in everyday interactions where experiences must be recalled and relayed to others (47). Narrative retellings in typical development have been found to focus on the gist of the original story (e.g., general explanation of the setting, such as a school) (9), while in ASD are more focused on local details of the story (e.g., specific objects in the physical setting, such as a chair) (41), suggesting that autistic individuals may have difficulty with central coherence (i.e., not as successfully convey the “big picture” takeaway of a story) and may have difficulty identifying the most relevant aspects of the story. Work in ASD has found that narrative ability is indeed more impacted in contexts with less structure (e.g., without the use of visual aids and a greater reliance on free recall) compared to more structured narrative contexts with concurrent visual supports (26, 27, 38). However, most studies using a narrative retelling context have not directly compared contexts (24, 33, 34, 39–46), making it difficult to determine how context affects narrative quality and infer neuropsychological mechanisms that may contribute to context effects.

Studies examining first-degree family members of autistic individuals constitute a useful approach to identify potentially heritable traits of ASD that might aggregate in more subtle form in biological relatives and be linked to autism-related genetic influence (50–56). Subclinical ASD-related personality and social-communication traits are reported at elevated rates among parents of autistic individuals (“ASD parents”), referred to as the broad autism phenotype (BAP) (51, 57–62). Additionally, differences in narrative ability have been observed among ASD parents compared to parents of children with typical development (28, 38, 63) and Down syndrome (63), suggesting possible genetic links to storytelling abilities.

Studies of pragmatic language in siblings of autistic individuals (“ASD siblings”) have drawn inconsistent conclusions, with some studies reporting pragmatic language deficits (64–67), and others identifying no differences in pragmatic ability (68, 69). Variability in prior studies may stem from differences in methodological approaches—such as parent-report questionnaires versus experimenter ratings, age ranges spanning toddlerhood to school-age, the specific domains of pragmatic language assessed, and variations in sample sizes and analytic methods (64–69). A recent meta-analytic study identified a small effect of poorer pragmatic language abilities in ASD siblings compared to controls (70). Interestingly, two studies using examiner ratings of pragmatic language in school-age children found a step-wise pattern, with autistic individuals demonstrating more difficulty with pragmatic language compared to their unaffected siblings, and siblings demonstrating more difficulty than controls (65, 66). The other two studies that observed differences did not include an ASD group for comparison, though findings indicated that the ASD sibling group demonstrated poorer pragmatic language abilities compared to the control group (64, 67). Differences in pragmatic language abilities may be more likely to emerge in unstructured, naturalistic tasks compared to standardized assessments in siblings (65), underscoring the importance of examining language abilities across contexts. To our knowledge, only one study to date has examined narrative ability in ASD siblings (28), highlighting a significant gap in the current literature for narrative ability ***—*** a specific pragmatic language skill which is well-studied in ASD and typical development.

A prior study by Nayar et al. (28), examining a First Telling narrative task in the same participants included here, found that autistic individuals used fewer descriptions of characters’ thoughts and feelings and inferred fewer causal relationships compared to controls, consistent with prior literature (23, 27, 29–31). Autistic individuals were also more likely to omit a key element of the story (e.g., setting, part of the plot, resolution) compared to controls, though no patterns emerged reflecting any specific story elements being less likely to be included than others. Narrative performance in ASD siblings partially mirrored patterns observed in the ASD group, with both groups using fewer descriptions of affect, cognition, and causal explanations than the control group. Unique to the ASD sibling group was the finding that they were less likely to establish the story setting than the ASD and control groups, suggesting they may have difficulty initiating the story and grounding their narrative in the global context. Although narrative skills were largely similar between parents of autistic and non-autistic individuals, some subtle differences emerged. Differences were only observed in the *types* of inferred causal relationships, but no differences emerged in the overall amount of inferred causal relationships or mentions of emotional and cognitive internal states of characters across the storybook. ASD parents provided more frequent explanations of the storybook character’s affect and cognition (e.g., why the character felt happy), while parents of typically developing individuals more often included causal descriptions of the character’s behavior (e.g., why the character looked behind a rock). The authors interpreted these findings to suggest that while narrative differences are evident in autistic individuals and their siblings, parents show more subtle differences in their storytelling approaches. This study expanded on these findings to examine narrative recall in this same participant sample and explored patterns of similarities and differences in ASD and first-degree relatives in this more challenging narrative task.

Visual attention influences how an individual interprets the environment and relates to general cognition (71, 72), language processing (73–75), and sensory regulation (76, 77). Differences in social attention have been observed in individuals with ASD, their parents, and siblings, including reduced attention to social stimuli (4, 28, 78–93), with social attention being the most impacted in ASD when viewing scenes with higher social saliency (74). However, some studies in infant siblings have found increased social attention compared to controls (94) or no differences (95). Importantly, studies to date have examined visual attention to social and nonsocial stimuli primarily in infant siblings, rather than children and adults. In research utilizing concurrent eye-tracking and narrative elicitation, evidence suggests that different attentional styles in autistic individuals and their parents relate to differences observed in narrative (28, 38), suggesting that what participants pay attention to influences what they speak about, and vice versa, and that groups may capitalize on different types of visual information to inform their narratives. In two prior studies using the same sample of autistic individuals, controls, and parents, and the same First Telling narrative context as the present study, individuals with ASD were found to attend less to the setting than controls, while no differences were seen between parent groups (28, 38). Nayar et al. (28) also included a group of ASD siblings, and found patterns that mirrored those observed in ASD, showing less attention to the setting. Individuals with ASD and their siblings also tended to not explore the entire visual scene, and mostly attended to similar groups of stimuli (e.g., setting information, characters), whereas the control group explored more diverse types of visual information. Findings were interpreted as support that differences in visual attention could contribute to the narrative patterns observed in autistic individuals and their siblings, highlighting the role of attention in shaping how individuals process and convey stories. Consistent with this interpretation, Mangnus et al. (96) recently reported that pupillary and brain responses during a viewing of an animated clip were less variable in autistic individuals compared to those without autism, perhaps suggesting that the information was processed in a more local or fixed manner (as opposed to the broader narrative context), providing additional evidence for more detailed attentional and cognitive patterns.

This study builds directly on this prior work by examining narrative in a less-structured context where participants retell a story (Retell task) that was initially narrated from a wordless picture book (First Telling). The Retell task context induces increased cognitive demands by requiring individuals to draw on memory (e.g., encoding, storage, retrieval) to reconstruct how elements of the story were meaningfully connected—more closely mirroring naturalistic storytelling contexts and challenges therein.

Previously published data on First Telling narratives (28) and visual attention during the narrative (28, 38) were included in the present study solely for comparison with the Retell task and were not otherwise re-reported. Narrative data for the present study were analyzed by applying the same hand-coding scheme that was used with First Telling narratives in Nayar et al. (28) to Retell narratives to inform a direct comparison. Narrative retell ability was also examined in relationship to visual attention patterns measured during the First Telling to explore whether visual attention to different aspects of the picture book (e.g., characters, background) was related to how participants later structured their narratives, potentially serving as an index of initial narrative encoding strategies.

We predicted that all groups would produce more sophisticated narratives in the structured First Telling task, due to increased cognitive demands in the Retell task, consistent with prior literature demonstrating that less structured discourse contexts pose greater challenges to autistic groups (26, 27, 38). The ASD group was expected to show the largest decline in narrative quality from the First Telling to Retell, while ASD siblings and parents were predicted to show similar patterns of narrative differences as the ASD group but with a more attenuated pattern of decline compared to the ASD group. Second, we predicted that visual attention patterns in the First Telling would relate to quality of the Retell narratives for each group. Given that groups differed in their visual attention during the initial viewing of the picture book (28, 38), they may have initially encoded distinct visual elements of the storybook that impacted the quality of narrative retellings.

## 2 Methods

### 2.1 Participants

Participants included 54 individuals with ASD, 41 ASD siblings, 156 ASD parents, 49 controls without a personal or family history of ASD, and 59 parent controls. See **Table 1** for demographic information. All participants had full-scale IQs (FSIQ) and verbal IQs (VIQ) >80, as measured by the Weschler Intelligence Scale for Children-Fourth Edition (WISC-IV) for children under 16 years of age or the Weschler Abbreviated Scale of Intelligence (WASI) for individuals over 16 years of age (97–99), and had verbal spoken language consistent with modules 3 or 4 of the ADOS-2. Participants were at least 10 years of age. This age range was selected because the ability to produce complex narratives is typically well-established by this age (3, 49, 100–102). All participants spoke English as a primary language and had no family history of genetic conditions related to ASD, such as fragile X syndrome. Exclusionary criteria for control participants included a family history of ASD and language impairment. Recruitment was focused throughout the Midwest region using registries, clinics, advocacy groups, schools, community events, and advertisements. Efforts were made to recruit intact families. However, some parents participated without their child, some children participated without their parent, and in some parent-child dyads, child data may have been excluded due to study exclusion criteria. All study materials were approved by the Northwestern University Institutional Review Board.

**Table 1:** Demographic information.

Demographic Information and Word Count	ASD	ASD Siblings	Controls	ASD Parents	Parent Controls
	M(SD) Range	M(SD) Range	M(SD) Range	M(SD) Range	M(SD) Range
N (M/F)^a,b^	54 (46/8)	41 (15/26)	49 (25/24)	156 (60/96)	59 (23/36)
Age (years)^c^	18.62 (6.18) 10.02-35.21	17.16 (4.20) 10.63-29.39	19.04 (5.34) 10.53-33.25	46.31 (8.09) 27.74-66.42	42.51 (10.27) 22.94-63.89
FSIQ^a,b^	106.57 (12.83) 80-131	115.51 (11.38) 89-134	117.71 (11.99) 89-142	111.51 (11.58) 82-136	114.98 (11.49) 86-139
VIQ^a,b^	106.41 (13.78) 84-146	116.12 (13.55) 82-146	118.77 (11.72) 93-142	109.98 (11.43) 80-132	111.92 (12.83) 82-138
PIQ^a,b,c^	104.26 (15.43) 68-131	112.41 (11.96) 86-141	113.52 (14.03) 79-143	109.96 (12.05) 72-137	113.63 (11.70) 86-148
Word Count First Telling	414.05 (171.36) 188-1029	410.95 (177.85) 171-1160	407.78 (143.39) 196-854	482.24 (194.84) 203-1165	468.30 (152.66) 210-909
Word Count Retell	247.17 (161.50) 39-829	276.49 (182.44) 52-960	283.22 (162.42) 69-890	344.00 (161.70) 106-1046	339.20 (128.70) 95-711
Race	N (%)	N (%)	N (%)	N (%)	N (%)
White	36 (66.7%)	31 (75.6%)	35 (71.4%)	110 (70.5%)	55 (93.2)
Black/African American	2 (3.7%)	1 (2.4%)	3 (6.1%)	9 (5.8%)	0 (0%)
Asian	1 (1.9%)	1 (2.4%)	9 (18.4%)	3 (1.9%)	1 (1.7%)
Native Hawaiian/Pacific Islander	0 (0%)	0 (0%)	0 (0%)	1 (0.6%)	0 (0%)
Biracial	2 (3.7%)	0 (0%)	1 (2.0%)	2 (1.3%)	0 (0%)
Other	1 (1.9%)	0 (0%)	1 (2.0%)	0 (0%)	0 (0%)
Not reported	12 (22.2%)	8 (19.5%)	0 (0%)	31 (19.9%)	3 (5.1%)
Ethnicity						
Hispanic	0 (0%)	0 (0%)	1 (2%)	6 (3.8%)	3 (5.1%)
Non-Hispanic	11 (20.4%)	16 (39%)	48 (98%)	132 (84.6%)	49 (83.1%)
Not reported	43 (79.6%)	25 (61%)	0 (0%)	18 (11.5%)	7 (11.9%)

a significant difference between ASD and Control Groups.

b significant difference between ASD and ASD Sibling Groups.

c significant difference between ASD Parent and Parent Control Groups.

For the ASD group, in addition to a prior clinical diagnosis, the Autism Diagnostic Observation Schedule-2 (ADOS-2) (103) and/or the Autism Diagnostic Interview, Revised (ADI-R) (104) was used to confirm a diagnosis of ASD. To rule out ASD in siblings of individuals with ASD and controls, participants were screened for medical history and the ADOS-2 (103) was administered. Individuals who met criteria for ASD based on the ADOS-2 were excluded from the present study.

### 2.2 Narrative elicitation tasks

#### 2.2.1 First telling

Participants narrated a 24-page picture book, *Frog Where Are You?* (105), page-by-page while viewing the stimulus on an eye tracker. This picture book is about a boy and his pet dog as they search for their missing frog, and it has been used extensively in the narrative literature (e.g., 24, 26, 27, 38, 39, 106–112). Procedures for administration of the First Telling were consistent with Lee et al. (38) and Nayar et al. (28), and First Telling narrative and eye tracking results were previously reported in Nayar et al. (28) for all groups.

#### 2.2.2 Retell

After a delay of approximately 10 minutes, participants were asked to retell their narrative from the First Telling task to an examiner. During the delay, participants completed another task where they named arrays of familiar items as part of the larger research battery. Participants were instructed to start at the beginning of the story and tell the examiner all they could remember. No visual cues were provided, thus no data were collected from the eye tracker because there was not visual stimuli.

### 2.3 Transcription

All narratives were transcribed verbatim from video by transcribers blind to group status using Systematic Analysis of Language Transcripts (SALT) (113) conventions. Both ELAN version 5.8 (114) and SALT were used as transcription software dependent on other needs for the data within the broader project through which these data were collected (e.g., use of transcripts synchronized with audio). Transcribers were trained to ≥80% training reliability on a minimum of 3 consecutive files on word-word agreement. Twenty nine percent of all Retell files were randomly selected with similar representation across groups for word-by-word reliability, and mean reliability was 95%. Transcripts were then processed through Linguistic Inquiry Word Count (LIWC) (115) to determine word count, excluding utterances spoken by the examiner, reformulations, and transcription notations and symbols.

### 2.4 Hand coding

The narrative coding scheme applied to First Telling and Retell narratives was adapted from prior work (3, 27, 110, 116, 117) and examines several key domains of narrative quality, as outlined below. The coding scheme was also applied in Nayar et al. (28).

#### 2.4.1 Story structure

The presence of descriptions of key story elements were coded across the narrative, including the setting, plot instantiation, five search episodes, and resolution. Each present key story element was tallied, and scores were summed to create a total score for *Story Components*, with a range of possible scores from 0-8.

**Setting.** Descriptions of the initial story setting, such as the physical or temporal setting.

**Plot Establishment.** Mention of the story’s central search theme (i.e., the frog’s escape).

**Search Episodes.** Each of the story’s five central search episodes were assessed for presence. See **Table 2** for descriptions of each story episode.

**Resolution.** Description of the frog being found.

**Table 2:** Descriptions of the 5 search episodes.

Search Episode	Event
1	The boy and the dog search for the frog in the bedroom, and the dog falls out the window.
2	The boy looks in a hole for the frog and finds a gopher. The dog is chased by bees.
3	The boy searches for the frog in a tree and is chased by an owl.
4	The boy finds a deer, rides the deer with the dog, and they fall over a cliff.
5	The boy and dog land in a pond and look for the frog.

#### 2.4.2 Thematic coherence

The establishment and maintenance of the theme were coded.

**Theme Establishment**. Initial description that the frog was missing or that the boy and dog were searching for the frog.

**Theme Maintenance**. Any additional description throughout Search Episodes 1–5 that the boy and dog were searching for the frog.

#### 2.4.3 Evaluation

The use of evaluative devices in the narrative was assessed by examining descriptions of *Affect and Cognition* and *Causal Explanations*.

**Affect and Cognition.** Descriptions of *Affective States and Behaviors* and *Cognitive States and Behaviors* (see below) were summed and examined as percent of total word count. Additionally, *Affective States and Behaviors* and *Cognitive States and Behaviors* were examined as a percent of all descriptions of *Affect and Cognition* to assess the proportion of usage of affect compared to cognition.

***Affective States and Behaviors*.** Descriptions of character’s emotions (e.g., happy, sad, scared) and behaviors related to their emotional states (e.g., smile, cower, kiss) were tallied.

***Cognitive States and Behaviors.*** Descriptions of character’s thoughts and internal states (e.g., think, realize) and behaviors related to those cognitive states (e.g., escape, sneak, hide) were tallied.

**Causal Explanations.** Descriptions of *Causal Explanations of Behaviors* and *Causal Explanations of Affect and Cognition* (see below) were summed and examined as a percent of total word count. Causal explanations were coded for clear language used to denote causality, such as “because”. More subtle uses of causal language were also included, such as language markers including “so”, “since”, “as a result”, “in order to”, and “therefore”. Additionally, *Causal Explanations of Behaviors* and *Causal Explanations of Affect and Cognition* were examined as a percent of all descriptions of *Causal Explanations* to assess the proportion of the types of causality used by participants.

***Causal Explanations of Behaviors.*** Descriptions of the cause or motivation of a behavior were tallied. For example, the following descriptions were coded:

“The boy was climbing to look in a tree because the frog could be there.”

“The dog ran by the tree in order to get away from the owl.”

***Causal Explanations of Affect and Cognition.*** Descriptions of the cause or motivation of an emotion or thought were tallied. For example, the follow descriptions were coded:

“The boy was worried because the frog was lost.”
“The dog was thinking that he should get away from the bees.”

#### 2.4.4 Other codes

Narratives were additionally assessed for the presence of excessive detail and topic perseveration. Excessive detail was rated subjectively by coders by classifying utterances as ‘excessive’ or ‘not excessive’. Topic perseveration was coded when there were 3 or more mentions of one topic throughout the narrative.

For example, the follow utterances throughout one transcript were coded as topic perseveration:

“Okay I was already wondering what the big boots were about.”
“Now he’s looking in the big boots which look too big for him.”
“Wow those boots are way too big for him.”
“What is with the boots?”
“I still can’t get over those boots those are so funny.”

### 2.5 Coding reliability

All files for the First Telling and Retell tasks were coded from the transcripts (described above) by coders blind to group status. Coders were trained to ≥80% training reliability on each code with an even representation from groups. Thirty-six percent of First Telling and 22% of Retell files were double coded for reliability. A larger sample of files were chosen for reliability in the First Telling given that it was the first application of the coding system, to ensure consistency across coders. Categorical variables were assessed using percent agreement, and continuous variables were assessed using intraclass correlation coefficients (ICCS; 118). ICCs from.5-.75 represent moderate agreement,.75-.9 represent good agreement, and >.9 represents excellent agreement (119). Percent agreement for categorical variables met or exceeded 80% across groups, except in the ASD group (76% for First Telling and 61% for Retell) and control group (75% for First Telling) for ratings of presence of setting. ICCs for continuous variables were greater than.76, indicating good to excellent agreement across codes.

### 2.6 Gaze procedures

Eye tracking was collected during the First Telling task on a Tobii T60 eye tracker. Gaze group differences have previously been published (28, 38). In the present paper, the principal component analyses (PCA) reported in Nayar et al. (28) from the First Telling were used for correlational analyses with Retell narratives. The PCA included variables characterizing the percentage of fixations to social (animate areas of interest; i.e., characters) and nonsocial (inanimate areas of interest; i.e., setting) stimuli, percentage of perseverative and regressive fixations within social and nonsocial stimuli, and percentage of fixation transitions between social and nonsocial stimuli. Higher scores on the PCA component indicated greater visual attention to social stimuli.

### 2.7 Analysis plan

#### 2.7.1 Group differences

For continuous narrative variables, a series of 2 x 2 (group x narrative context) repeated-measure analysis of covariance (ANCOVA) were conducted for ASD, ASD sibling, and control groups, and repeated-measure analysis of variance (ANOVA) were applied for parent groups. Simple effects were assessed following significant or marginally significant interaction effects or main effects of group given the subclinical nature of the BAP and heterogeneity observed in ASD. For independent samples (i.e., parent groups, ASD compared to controls, ASD siblings compared to controls), one-way ANCOVAs were conducted. For dependent samples (i.e., ASD compared to ASD siblings), a series of linear mixed effects regression models were conducted with family included as a random effect using the lmer (120) package for R (R version 3.6.1). The presence or absence of narrative variables was analyzed using 2 x 2 chi-square tests or a Fisher’s exact test in instances where expected cell values were <5.

**Covariates.** When statistically possible to include a covariate, VIQ was included due to significant differences between the ASD group and the ASD sibling and control groups (**Table 1**), and known relations between language abilities and narrative in children (121). Groups were not significantly different on age or story length, as measured by word count; thus, these variables were not included as covariates. The autistic group did have a skewed sex ratio compared to other groups, with females being significantly less represented than males, though no sex differences were observed on the summary narrative variables (i.e., *Story Components, Affect and Cognition, Causal Explanations*) within the ASD group (*p*s >.78). Given no sex differences on summary narrative variables and a small sample of females in the autistic group (n = 8), sex was not included as a covariate. No covariates were applied to chi-square or Fisher’s exact test models due to limitations of the models. No covariates were included in parent analyses.

#### 2.7.2 Relationships between first telling and retell tasks

A series of linear mixed effect models were conducted within each group, investigating the effect of First Telling narrative variables on the same variable in the Retell task. The lme4 package (120) was used to fit the model using R statistical software (version 3.6.1). Standardized beta values from the regression models were calculated. Models accounted for random effects within each individual.

#### 2.7.3 Relationships between narrative quality in retell task and gaze

Exploratory Pearson correlations were conducted within each group to assess relationships between the main narrative variables (i.e., *Story Components, Affect and Cognition, Causal Explanations)* and the eye tracking component variable derived from the PCA.

#### 2.7.4 Multiple comparisons

The Benjamini-Hochberg procedure (122) was used to adjust *p*-values for multiple comparisons in analyses using a false discovery rate of 0.10. Due to the novel investigation of narrative retelling in first-degree relatives without clinical diagnoses, as well as the potentially subtle nature of group-level differences in this subclinical population and heterogeneity seen in ASD, both Benjamini-Hochberg corrected and uncorrected *p*-values are reported to reduce Type 1 and Type 2 error, respectively. While correction for multiple comparisons reduces the risk of Type 1 error, findings that did not withstand corrections may still demonstrate meaningful patterns in subtle narrative differences. This approach provides a transparent and comprehensive view of the data while balancing sensitivity to potentially meaningful effects.

## 3 Results

### 3.1 Narrative differences across groups and contexts

**Figure 1:**
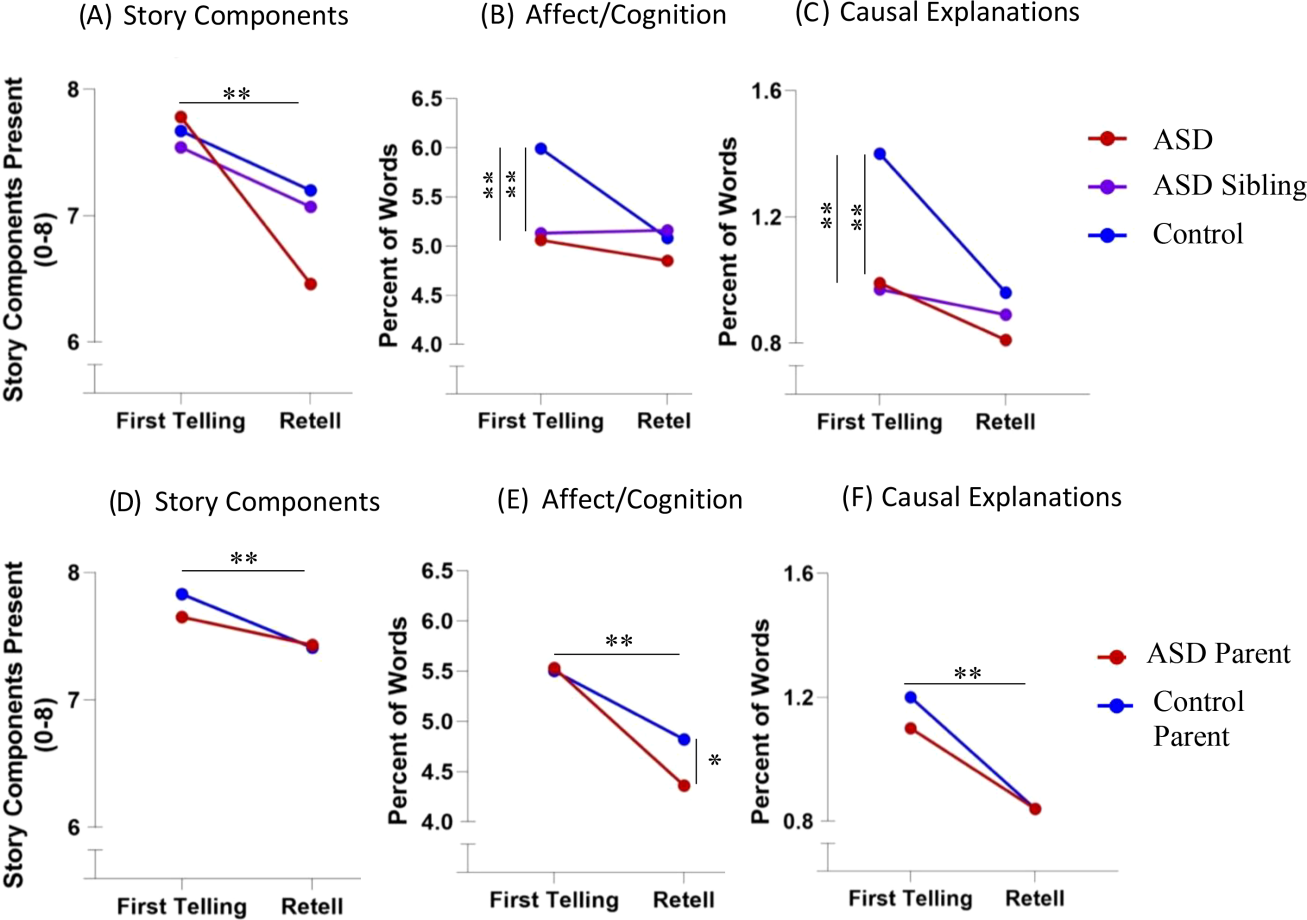
Narrative patterns depicting **(A)** Number of key elements included in the narratives of the ASD, ASD Sibling, and Control groups; **(B)** Frequency of descriptions of the character’s thoughts and feelings in the ASD, ASD Sibling, and Control groups; **(C)** Frequency of descriptions of causal explanations of affect, cognition, or behavior in the ASD, ASD Sibling, and Control groups; **(D)** Number of key elements included in the narratives of ASD Parent and Control Parent groups; **(E)** Frequency of descriptions of the character’s thoughts and feelings in the ASD Parent and Control Parent groups; **(F)** Frequency of descriptions of causal explanations of affect, cognition, or behavior in the ASD Parent and Control Parent groups. ** indicates p < .01.

Mean and standard deviations are reported in **Table 3** and results are summarized below. See **Figure 1**.

**Table 3:** Descriptive Statistics for Repeated Measures ANCOVA.

Variables	ASD		ASD Siblings		Controls		ASD Parents		Parent Controls	
	First Telling	Retell	First Telling	Retell	First Telling	Retell	First Telling	Retell	First Telling	Retell
	M (SD)	M (SD)	M (SD)	M (SD)	M (SD)	M (SD)	M (SD)	M (SD)	M (SD)	M (SD)
Story Components Present	7.78(.42)	7.54(.78)	7.07(1.42)	6.46(2.03)	7.67(.88)	7.20(1.24)	7.65(.68)	7.43(.87)	7.83(.42)	7.41(.89)
Affect/Cognition^a^	5.06(1.42)	5.13(1.27)	5.16(1.83)	4.85(1.74)	5.99(1.41)	5.08(1.24)	5.53(1.51)	4.36(1.05)	5.50(1.45)	4.82(1.39)
Causal Explanations^a^	.99(.70)	.97(.60)	.89(.95)	.81(.68)	1.40(.64)	.96(.68)	1.10(.59)	.84(.56)	1.20(.69)	.84(.53)
Causal Explanations of Affect/Cognition^b^	55.61(29.05)	45.07(33.46)	49.71(38.91)	34.80(34.20)	53.76(25.24)	37.76(36.90)	53.99(29.39)	62.56(32.41)	62.35(24.89)	68.26(32.38)
Causal Explanations of Behavior^b^	44.39(29.05)	54.93(33.46)	50.29(42.77)	65.20(34.20)	46.24(25.24)	62.24(36.90)	46.01(29.39)	37.33(32.41)	37.65(24.89)	31.74(32.38)
Affective States and Behaviors^c^	19.02(11.04)	19.27(9.11)	15.84(13.43)	15.08(14.45)	19.02(7.94)	12.92(10.32)	21.05(10.17)	19.15(12.35)	19.34(8.61)	18.08(10.15)
Cognitive States and Behaviors^c^	80.98(11.04)	80.73(9.11)	84.16(13.43)	84.92(14.45)	80.98(7.94)	87.08(10.32)	78.95(10.17)	80.85(12.35)	80.66(8.61)	81.92(10.15)

a Variables are calculated as a percent of total word count.

b Variables are calculated as a percent of Causal Explanations.

c Variables are calculated as a percent of Affect/Cognition.

#### 3.1.1 ASD vs. control groups

There was a significant interaction effect between diagnostic group and context for *Story Components* (*F*(1, 100) = 4.66, *p* = .033, ηp^2^ = .04, adjusted *p* = .223), with both groups including a similar number of story components in the First Telling (*F*(1, 102) = 0.56, *p* = .446, ηp^2^ = .01, adjusted *p* = .741), and the ASD group using marginally fewer story components in the Retell task (*F*(1, 100) = 2.92, *p* = .091, ηp^2^ = .03, adjusted *p* = .443). Main effects of group emerged for *Affect and Cognition* (*F*(1, 100) = 7.99, *p* = .006, ηp^2^ = .07, adjusted *p* = .069), and *Causal Explanations* (*F*(1, 100) = 3.99, *p* = .049, ηp^2^ = .04, adjusted *p* = .306), with the autistic group using fewer descriptions in the First Telling than the control group (Affect and Cognition: *F*(1, 102) = 12.51, *p* = .001, ηp^2^ = .11, adjusted *p* = .020; Causal Explanations: *F*(1, 102) = 7.09, *p* = .009, ηp^2^ = .07, adjusted *p* = .089), and there were no differences in the Retell task (Affect and Cognition: *F*(1, 100) = 1.66, *p* = .201, ηp^2^ = .02, adjusted *p* = .578; Causal Explanations: *F*(1, 100) = 0.46, *p* = .500, ηp^2^ = .01, adjusted *p* = .779). In the Retell task, the ASD group was more likely to omit the setting (*χ* ^2^(1) = 14.19, *p* <.001, adjusted *p* = .020) and search episode 5 (*χ* ^2^(1) = 5.35, *p* = .021, adjusted *p* = .170) compared to the control group. When examining the number of participants who excluded a description of the setting in the Retell task, 29.6% of the autistic group (16 participants) omitted the setting compared to only 2% of the control group (1 participant).

#### 3.1.2 ASD vs. ASD sibling groups

A main effect of narrative context emerged for *Story Components* (*F*(1, 92) = 9.59, *p* = .003, ηp^2^ = .09, adjusted *p* = .046), with both groups including fewer *Story Components* in the Retell task compared to the First Telling. There was a significant group by context interaction effect for *Causal Explanations of Behavior* (*F*(1, 65) = 6.37, *p* = .014, ηp^2^ = .09, adjusted *p* = .121) and *Causal Explanations of Affect and Cognition* (*F*(1, 65) = 6.37, *p* = .014, ηp^2^ = .09, adjusted *p* = .121), characterized by the ASD group using more *Causal Explanations of Behavior* in the Retell task, and the opposite pattern emerging in the ASD sibling group, though simple effects within tasks were not significant (unadjusted *p*s >.09). There was a main effect of context for *Affective States and Behaviors* (*F*(1, 92) = 12.18, *p* <.001, ηp^2^ = .12, adjusted *p* = .020) and *Cognitive States and Behaviors* (*F*(1, 92) = 12.18, *p* <.001, ηp^2^ = .12, adjusted *p* = .020), revealing that both groups used more descriptions of affect in the First Telling task and more descriptions of cognition in the Retell task.

#### 3.1.3 ASD sibling vs. control groups

A main effect of narrative context emerged for *Story Components* (*F*(1, 87) = 12.38, *p* <.001, ηp^2^ = .13, adjusted *p* = .020), with both groups including fewer *Story Components* in the Retell task compared to the First Telling. There was a significant interaction effect between group and narrative context for *Affect and Cognition* (*F*(1, 87) = 7.53, *p* = .007, ηp^2^ = .08, adjusted *p* = .074), with the ASD sibling group using significantly less *Affect and Cognition* in the First Telling than the control group (*F*(1, 88) = 9.77, *p* = .002, ηp^2^ = .10, adjusted *p* = .035), while groups used comparable amounts of *Affect and Cognition* in the Retell task [*F*(1, 87) = 0.03, *p* = .868, ηp^2^ = .00, adjusted *p* = 1.007]. Though the group by context interaction was only marginal for *Causal Explanations* (*F*(1, 87) = 3.92, *p* = .051, ηp^2^ = .04, adjusted *p* = .306), simple effects revealed a similar pattern to *Affect and Cognition*, with the ASD sibling group using significantly fewer descriptions of *Causal Explanation* in the First Telling compared to the control group (*F*(1, 88) = 10.78, *p* = .001, ηp^2^ = .11, adjusted *p* = .020) and no differences in the Retell task (*F*(1, 87) = 0.07 *p* = .795, ηp^2^ = .00, adjusted *p* = .967). There was a significant interaction effect for *Causal Explanations of Behaviors* (*F*(1, 73) = 4.66, *p* = .034, ηp^2^ = .06, adjusted *p* = .223) and *Causal Explanations of Affect and Cognition* (*F*(1, 87) = 4.66, *p* = .034, ηp^2^ = .06, adjusted *p* = .223), characterized by the control group using more *Causal Explanations of Affect and Cognition* in the First Telling task and more *Causal Explanations of Behavior* in the Retell task, with the opposite pattern emerging in the ASD sibling group, though differences within tasks did not meet thresholds for significance (*F*(1, 88) = 1.08, *p* = .301, ηp^2^ = .01, adjusted *p* = .716). A main effect of context for *Affective States and Behaviors* (*F*(1, 87) = 8.08, *p* = .006, ηp^2^ = .09, adjusted *p* = .069) and *Cognitive States and Behaviors* (*F*(1, 87) = 8.08, *p* = .006, ηp^2^ = .09, adjusted *p* = .069) revealed that both groups used more descriptions of affect in the First Telling task and more descriptions of cognition in the Retell task. The ASD sibling group was significantly less likely to include the setting in the Retell task compared to the control group (*p* = .001, adjusted *p* = .020).02% of the control group (1 participant) omitted the setting compared to 21.9% of the ASD sibling group (9 participants).

#### 3.1.4 ASD parent vs. control parent groups

A significant interaction emerged for *Affect and Cognition* [*F*(1, 213) = 4.29, *p* = .040, ηp^2^ = .02, adjusted *p* = .245], where groups used similar amounts of *Affect and Cognition* in the First Telling (*F*(1, 220) = .001, *p* = .974, ηp^2^ = .00, adjusted *p* = 1.00), and *post-hoc* tests revealed that the parent control group used significantly more *Affect and Cognition* in the Retell task than ASD parents (*F*(1, 213) = 6.71, *p* = .010, ηp^2^ = .03, adjusted *p* = .118). *Story Components* (*F*(1, 213) = 19.19, *p* <.001, ηp^2^ = .08, adjusted *p* = .002) and *Causal Explanations* (*F*(1, 213) = 33.78, *p* <.001, ηp^2^ = .14, adjusted *p* = .002) had a main effect of context, where both groups included more *Story Components* and *Causal Explanations* in the First Telling than Retell task. A main effect of context emerged for *Causal Explanations of Behaviors* (*F*(1, 181) = 6.43, *p* = .012, ηp^2^ = .03, adjusted *p* = .118), and *Causal Explanations of Affect and Cognition* (*F*(1, 181) = 6.43, *p* = .012, ηp^2^ = .03, adjusted *p* = .118), characterized by both groups using more *Causal Explanations of Affect and Cognition* in the First Telling and more *Causal Explanations of Behavior* in the Retell task.

### 3.2 Relationships between first telling and retell tasks

Results are reported in **Table 4** and are summarized below.

**Table 4:** Associations between First Telling and Retell Task.

First Telling	Group	Retell					
		Story Components		Affect/Cognition		Causal Explanations	
		*p*	β	*p*	β	*p*	β
Story Components	ASD	**.012**	**.32**	.100	.15	.675	-.06
	ASD Siblings	.026	.31	.694	-.04	.934	-.01
	Controls	**<.001**	**.53**	.068	.21	.080	-.26
	ASD Parents	.012	.21	.797	-.01	.878	-.01
	Parent Controls	.704	-.05	.276	-.13	.471	-.09
Affect/ Cognition	ASD	.934	.01	**<.001**	**.66**	**.006**	**.45**
	ASD Siblings	.209	.17	**<.001**	**.73**	.063	.32
	Controls	.109	.23	**<.001**	**.61**	.458	.12
	ASD Parents	.170	.12	**<.001**	**.61**	.195	.12
	Parent Controls	.200	.02	**.004**	**.39**	.234	.26
Causal Explanations	ASD	**.006**	**.41**	.122	.16	.744	.05
	ASD Siblings	.610	.07	.467	-.08	.254	.18
	Controls	.134	-.21	.622	-.06	.075	.29
	ASD Parents	.341	.08	.739	.02	.015	.21
	Parent Controls	.269	.21	.125	.20	.013	.34

Bolded values indicate that the finding withheld Benjamini Hochberg corrections.

β indicates standardized beta.

#### 3.2.1 ASD, ASD sibling and control groups

In all groups, *Story Components* in the First Telling predicted *Story Components* in the Retell task (ASD: *p* = .012, β = .32, adjusted *p* = .046; ASD Siblings: *p* = .026, β = .31, adjusted *p* = .088; Controls: *p* <.001, β = .53, adjusted *p* <.001), and *Affect and Cognition* in the First Telling predicted *Affect and Cognition* in the Retell task (ASD: *p* <.001, β = .66, adjusted *p* <.001; ASD Siblings: *p* <.001, β = .73, adjusted *p* <.001; Controls: *p* <.001, β = .61, adjusted *p* <.001). In autistic individuals, *Affect and Cognition* in the First Telling predicted *Causal Explanations* in the Retell task (*p* = .006, β = .45, adjusted *p* = .027), and *Causal Explanations* in the First Telling predicted *Story Components* in the Retell task (*p* = .006, β = .41, adjusted *p* = .026). Surprisingly, *Causal Explanations* in the First Telling did not predict *Causal Explanations* in the Retell task (unadjusted *p*s >.06).

#### 3.2.2 Parent groups

In ASD parents, *Story Components* in the First Telling predicted *Story Components* in the Retell task (*p* = .012, β = .21, adjusted *p* = .054), though this was not significant in the Parent Control group (*p* = .704, β = -.05, adjusted *p* = .831). In both parent groups, *Affect and Cognition* in the First Telling predicted *Affect Cognition* in the Retell task (ASD Parents: *p* <.001, β = .61, adjusted *p* = .002; Parent Controls: *p* = .004, β = .39, adjusted *p* = .036), and *Causal Explanations* in the First Telling predicted *Causal Explanations* in the Retell task (ASD Parents: *p* = .015, β = .21, adjusted *p* = .054; Parent Controls: *p* = .013, β = .34, adjusted *p* = .054).

### 3.3 Relationships between narrative retell and gaze

#### 3.3.1 ASD, ASD sibling, and control groups

There were no associations between narrative ability and the PCA-derived gaze component, where higher ratings on the PCA indicated more social visual attention (*rs* <.17, unadjusted *ps* >.37).

#### 3.3.2 Parent groups

There were no associations between narrative ability and the PCA-derived gaze component indicating social attention (*rs* > -.19, unadjusted *ps* >.15).

## 4 Discussion

The present study examined narrative abilities across contexts varying in structure among autistic individuals, ASD siblings, ASD parents, and respective control groups. Building on prior work, a detailed narrative hand coding scheme was applied to characterize narrative recall ability in autistic individuals and their first-degree relatives (i.e., parents and siblings). As previously discussed, little is known concerning the potential convergence of subclinical broader phenotypes in clinically unaffected siblings and parents, particularly in higher order social communication skills such as narrative and other pragmatic abilities, due to the focus on earlier development periods in the ASD sibling research. Results support prior literature identifying narrative differences impacted by context, while unexpectedly, they did not reveal an influence of visual attention on narrative retellings. Additionally, results demonstrated that patterns of narrative strengths and weaknesses in ASD were partially mirrored among siblings, while ASD parents resembled control parents, suggesting that narrative quality in the BAP may change throughout development.

This study examined which key story elements (i.e., setting, plot instantiation, search episodes, resolution) were omitted during the Retell task, and whether differential patterns emerged across groups. Findings from Nayar et al. (28) demonstrated that the ASD siblings were more likely to omit the setting in the First Telling compared to the ASD and control groups. Similarly, ASD siblings and individuals with ASD were more likely to omit the setting in the Retell task compared to controls, suggesting less attention to setting elements during the initial narrative, or differences in interpretation of such stimuli such that they were deemed less relevant to the overall story when producing the retell. Previous research examining setting establishment in narratives has been mixed in ASD. Studies with younger samples have reported no differences (39, 44, 123), while others with adult samples found reduced likelihood of mentioning the setting (41). Further examination of transcripts from the Retell task revealed that participants who omitted descriptions of the setting tended to skip ahead in the story, focusing on the story theme (i.e., search for the missing frog) or jumping directly into search-related activities (e.g., search episode 1, looking for the frog in the bedroom). For instance, two participants who did not establish the setting began their stories with, “the boy lost his frog”, and “the frog got outside”, without describing the temporal or physical setting/context of the story. A lack of attention to establishing the setting of the narrative in the retell result in limited narrative grounding for the listener, and may reflect challenges with narrative planning and organization (24, 124) among autistic individuals and siblings.

We predicted that the quality of narratives would decrease in the less structured recall context due to reduced visual support and increased cognitive demand, consistent with past work (26, 27, 38), and this pattern was confirmed in controls. For instance, individuals in the control group used more descriptions of affect and cognition in the more structured context (First Telling) and exhibited a decline in the less structured context (Retell). In contrast, autistic individuals and their clinically unaffected siblings produced narratives of similar quality across contexts, with diminished narrative quality in the ASD group appearing only in mention of key story components. This lack of decline across contexts may stem from between-group variability, as ASD and ASD sibling groups exhibited lower narrative quality than other groups in the structured context for descriptions of affect, cognition, and causal explanations. Indeed, this pattern of results aligns with findings from Losh and Capps (27), where autistic individuals used similar amounts of descriptions of affect, cognition, and causality across contexts. However, while controls in Losh and Capps (27) provided more of these descriptions in the less-structured context, the opposite pattern observed in the present study. These findings highlight that the control group may have modulated the form and content of their narrative depending on the demands of the discourse context, while the narratives from the ASD and ASD sibling groups were less context sensitive. The ASD and sibling groups may also have focused more on individual components of the story, while controls maintained the overall gist of the story, reflecting differences in central coherence.

This study found that all groups provided more descriptions of the character’s emotions in the First Telling and more descriptions of the character’s cognitive states in the Retell task, suggesting that the child groups initially focused on emotions but shifted towards cognitive states with repeated exposure. This pattern may reflect deeper processing during retelling, where greater attention is given to the characters’ thoughts. The lack of differences in parent groups may indicate that by adulthood, emotional and cognitive elements may be more integrated into a narrative. Additionally, this study examined the types of causal explanations linking a characters’ thoughts, emotions, or actions. Contrary to prior literature demonstrating reduced causal explanations in autistic individuals, this study found that autistic individuals and controls provided more causal explanations for behavior than ASD siblings, whereas ASD siblings offered more explanations for emotions and thoughts (23, 24, 27). This suggests that ASD siblings may have a relative strength in elaborating on emotional and cognitive states. Autistic individuals, particularly in the less structured Retell task, retained a strong grasp of the initial narrative and motivations behind characters actions. This ability to bridge the two contexts—applying causal reasoning in unstructured retelling—suggests a nuanced narrative capacity that may not be fully captured by studies focusing only on more structured tasks.

Finally, whereas prior work has shown that visual attention patterns are related to narrative quality in ASD and parents during simultaneous viewing and storytelling (28, 38), the current study found that no significant relationships were detected between social vs nonsocial visual attentional patterns during the First Telling and the content or quality of narratives during the Retell task in any group. It may be that gaze patterns captured during the First Telling may be too detached from the narrative content in the Retell task when analyzed using broad patterns of looking (e.g., time spent on social compared to non-social areas of interest). Similarly, a shift in attentional focus may have rendered initial gaze patterns less relevant for narrative recall. Additionally, the cognitive demands of the Retell task, which involve more memory retrieval and narrative organization, could diverge from the more immediate processing required during the First Telling. It is also possible that the gaze variables included were insufficient for capturing the most relevant attentional patterns related to narrative encoding as measured by our narrative retell coding scheme. To more precisely elucidate gaze-narrative relationships across contexts, future work should focus on more refined analyses of visual attention, such as synchronized analyses between language production and attentional focus.

## 5 Conclusions

In summary, findings contribute to literature on narrative ability in ASD by examining recall ability in relationship to first telling narratives and visual attention in ASD, siblings, and parents. These findings help to clarify the presentation of narrative ability across development and different first-degree relatives. Specifically, the study highlights a partially overlapping profile of narrative ability in individuals with ASD and their siblings, with both groups demonstrating similar patterns of narrative quality across contexts in contrast to controls who show the expected decline in narrative quality between structured and unstructured contexts. Results also underscore the importance of considering contextual factors in influencing narrative performance. Notably, while individuals with ASD and their siblings exhibited broadly comparable narrative profiles, siblings were more likely to include causal explanations for emotions and thoughts, pointing to a potential area of relative strength in the BAP. These findings suggest that the narrative ability in ASD siblings may not be as impacted as in individuals with ASD, but still reveals subtle differences that may reflect the broader genetic and developmental influences associated with ASD.

## 6 Limitations and future directions

Future work utilizing more naturalistic narrative contexts with increased social and cognitive demands, such as semi-structured conversations (e.g., 125, 126) may better capture how narrative ability is impacted in daily life and may reveal more nuanced subclinical differences in narratives among first-degree relatives of individuals with ASD. Longitudinal studies would be particularly valuable to understand how narrative abilities evolve across different developmental stages in both ASD and ASD sibling groups, particularly in light of the present findings highlighting some subtle differences in affective versus cognitive word use in child versus parent groups. Additionally, while this study was designed to explore familial patterns of narrative ability, similarities observed between groups may reflect not only shared genetic influences, but also shared environmental factors (e.g., parenting style). However, prior work has demonstrated that certain language traits are evident in parents even before having a child with autism (127), and early in development for siblings of autistic individuals (67, 70) and that language traits are associated with elevated polygenic risk for ASD (128), supporting a genetic influence on pragmatic skills. The present study was limited by a small sample size of females with ASD and thus sex differences were not examined. Given known sex-based narrative differences, such as increased use of descriptions of character’s thoughts and emotions in females (129, 130), it will be important for future work to consider differential patterns among males and females. The analyses in this manuscript were guided by *a priori* hypotheses across several theoretically- and empirically-motivated domains, requiring multiple comparisons both to replicate and expand upon prior work. Given the novel additions to this study—particularly examining the relationship between visual attention and narrative retelling in first-degree relatives without a clinical diagnosis—these findings represent an initial exploration of an understudied area. While statistical controls were applied (i.e., Benjamini-Hochberg corrections), the results warranted cautious interpretation, and replication in larger, independent samples is necessary to confirm the robustness and generalizability of these effects. Finally, future work examining narrative retellings should assess executive functioning skills (e.g., organization, planning, self-monitoring, working memory), learning and memory profiles (e.g., short term versus long-term visual and verbal memory), and attention to better elucidate the underlying neuropsychological processes that play a role in encoding, consolidating, and recalling stories.

## Data Availability

The data supporting the conclusions of this article will be made available by the authors upon request.
